# *Dirofilaria immitis* possesses molecules with anticoagulant properties in its excretory/secretory antigens

**DOI:** 10.1017/S0031182020000104

**Published:** 2020-04

**Authors:** Alicia Diosdado, Fernando Simón, Rodrigo Morchón, Javier González-Miguel

**Affiliations:** 1 Laboratory of Parasitology, Faculty of Pharmacy, University of Salamanca, Av. Licenciado Méndez Nieto s/n, 37007 Salamanca, Spain; 2 Laboratory of Parasitology, Institute of Natural Resources and Agrobiology of Salamanca (IRNASA-CSIC), C/ Cordel de Merinas 40-52, 37008 Salamanca, Spain; 3 Martsinovsky Institute of Medical Parasitology, Tropical and Vector Borne Diseases, Sechenov University, Malaya Pirogovskaya St. 20-1, 119435 Moscow, Russia

**Keywords:** Anticoagulant, coagulation cascade, *Dirofilaria immitis*, excretory/secretory antigens, factor Xa, serpin

## Abstract

*Dirofilaria immitis* is a parasitic nematode that survives in the circulatory system of suitable hosts for many years, causing the most severe thromboembolisms when simultaneous death of adult worms occurs. The two main mechanisms responsible for thrombus formation in mammals are the activation and aggregation of platelets and the generation of fibrin through the coagulation cascade. The aim of this work was to study the anticoagulant potential of excretory/secretory antigens from *D. immitis* adult worms (DiES) on the coagulation cascade of the host. Anticoagulant and inhibition assays respectively showed that DiES partially alter the coagulation cascade of the host and reduce the activity of the coagulation factor Xa, a key enzyme in the coagulation process. In addition, a *D. immitis* protein was identified by its similarity to the homologous serpin 6 from *Brugia malayi* as a possible candidate to form an inhibitory complex with FXa by sodium dodecyl sulfate polyacrylamide gel electrophoresis and mass spectrometry. These results indicate that *D. immitis* could use the anticoagulant properties of its excretory/secretory antigens to control the formation of blood clots in its immediate intravascular habitat as a survival mechanism.

## Introduction

*Dirofilaria immitis* is the filarial nematode that causes the animal cardiopulmonary dirofilariosis, a vector-borne zoonosis affecting mainly dogs and cats, and other carnivorous mammals throughout the world (Simón *et al*., 2012). One of the most striking characteristics of this parasitosis could be the ability of the adult worms to survive during years in their definitive location in the pulmonary artery and the right ventricle of the heart of suitable hosts, while apparently controlling the formation of serious thromboembolisms (González-Miguel *et al*., 2015). For that purpose, not only *Dirofilaria*, but other blood parasites have evolved developing anticoagulant strategies that interfere with the haemostatic system and that may result in survival mechanisms allowing parasites to evade host defence response in their intravascular habitat (Figuera *et al*., 2013; González-Miguel *et al*., 2016; Ayón-Núñez *et al*., 2018).

Anticoagulant mechanisms of the haemostatic system include inhibitors of primary (activation and aggregation of blood platelets) and secondary haemostasis (the coagulation cascade), as well as enhancers of the fibrinolytic activity of plasmin that leads to the lysis of the fibrin clot once it has been formed and, hence, for restoring the normal physiological state that guarantees correct vascular permeability (Mebius *et al*., 2013; Chapin and Hajjar, 2015). In this sense, the activation of the fibrinolytic system of the host mediated by the participation of excretory/secretory and surface-associated antigens from *D. immitis* has been widely demonstrated (González-Miguel *et al*., 2012, 2013), but no study on the possible capacity of this parasite to inhibit primary and/or secondary haemostasis has been published. Regarding the latter, the ability of several helminth parasites to excrete proteins capable of inhibiting the coagulation cascade of its host has already been described (Knox, 2007). Most of these molecules belong to the group of serpins, a highly conserved superfamily of enzymes whose action mechanism implies the formation of a very stable complex with its target protein (Molehin *et al*., 2012). Their inhibitory effects are produced against different proteins among which is the activated factor X (FXa), that has a pivotal role as a convergence point between the intrinsic and extrinsic pathways of the coagulation cascade and as a triggering enzyme of the conversion of fibrinogen into fibrin (Adams and Bird, 2009).

The aim of this work was to study the anticoagulant effect of excretory/secretory antigens from *D. immitis* adult worms (DiES) on the secondary haemostasis of its host, focussed on searching an inhibitory serpin of FXa, in order to suggest a novel mechanism of the parasite to avoid the appearance of thromboembolisms in its immediate intravascular habitat.

## Materials and methods

### Collection of the antigenic extract and blood samples

DiES were prepared as previously described (González-Miguel *et al*., 2012) with minor modifications. Live worms were obtained from naturally infected dogs and washed in sterile phosphate buffered saline solution (PBS), pH 7.2. After this, 15 male and 15 female adult worms were incubated in 50 mL of Eagle's minimum essential medium supplemented with 50 U mL^−1^ penicillin and 50 *μ*g mL^−1^ streptomycin for 24 h at 37°C. A cocktail of protease inhibitors was added to the medium (Maizels *et al*., 1991) and the homogenate was dialysed against water for 24 h shaking. After filtering the medium through an Amicon YC05 membrane (Millipore), the protein concentration of DiES products was measured by a DC protein assay commercial kit (Bio-Rad). The antigenic extract was tested for the presence of endotoxin contamination using a quantitative Limulus amebocyte lysate test (BioWhittaker), which showed that endotoxin quantity was under the sensitivity level of cell stimulation (<0.4 U mg^−1^ protein). DiES were stored at −80°C until their use.

Blood samples from healthy dogs were collected in tubes containing sodium citrate and centrifuged immediately at 1500 × ***g*** for 15 min. Plasma was separated from blood cells of the samples and stored at −80°C until its use. A pool of 10 samples was used to carry out all the following experiments. All the owners gave their consent to participate in this study.

### Anticoagulation assays

The anticoagulant activity of DiES was evaluated by three different coagulation times: the activated partial thromboplastin time (APTT), the prothrombin time (PT) and the thrombin time (TT). The APTT and the PT assays respectively measured the intrinsic and the extrinsic pathways of the coagulation cascade, as well as the common pathway, while the TT assay evaluated the conversion of fibrinogen to fibrin. All of them were carried out following the method described by Gan *et al*. (2009) with some modifications. For the APTT assay, 0.5 *μ*g per well of DiES were added to multiwell microplates (Costar) and incubated with 50 *μ*L of plasma and 50 *μ*L of APTT reagent (BioSystems) for 3 min at 37°C. After that, 50 *μ*L of CaCl_2_ (BioSystems) were added to each well to initiate the clotting reaction. For the PT assay, 0.5 *μ*g per well of DiES were incubated with 50 *μ*L of plasma for 2 min at 37°C, and then 100 *μ*L of PT reagent (BioSystems) were added to initiate the clotting reaction. For the TT assay, 0.5 *μ*g per well of DiES were incubated with 100 *μ*L of plasma. After incubating for 2 min at 37°C, 100 *μ*L of TT reagent (BioSystems) were added to the previous mixtures to initiate the clotting reaction. Prior to starting the assays, frozen plasma and the reagents were thawed at 37°C. In all cases, the formation of the clot was monitored by measuring the absorbance at 655 nm each 6 s during a period of 42 s in a Microplate Absorbance Reader iMark (Bio-Rad). PBS was used as negative control. Each sample was analysed in triplicate.

### Activated factor X inhibition assay

The inhibitory activity of DiES against FXa was evaluated using a chromogenic assay carried out in a total volume of 100 *μ*L in multiwell microplates (Costar) according to the methodology described by Gan *et al*. (2009) with some modifications. In each well, 0.1 *μ*g of DiES and the native human FXa (ThermoFisher Scientific) at a final concentration of 4 nm were incubated in HEPES-BSA buffer (50 mm Hepes, pH 7.5, 100 mm NaCl, 5 mm CaCl_2_, 1 mg mL^−1^ BSA) for 15 min at 37°C. After that, the chromogenic substrate S-2765 of FXa (Chromogenix) was added at a final concentration of 200 *μ*m and incubated for 2 h 30 min at 37°C. The hydrolysis of the substrate was monitored by measuring the absorbance at 415 nm every 30 min. PBS was used as negative control. Each sample was analysed in triplicate.

### Binding of DiES proteins to FXa

Since inhibitors of serine proteases trap its target protein into an irreversible complex (Gettins, 2002), a sodium dodecyl sulfate polyacrylamide gel electrophoresis (SDS-PAGE) was carried out following the protocol described by Fonseca *et al*. (2011) with some modifications to determine if FXa was capable of binding to some protein of DiES. Twenty micrograms of DiES, 1 *μ*g of FXa and a pre-incubated mixture of both compounds were loaded into 12% polyacrylamide gels. Previously, all samples were incubated in 5 mm Hepes buffer, pH 7.4, for 45 min at room temperature. After finishing the electrophoresis, gels were stained with Coomassie Blue.

### Mass spectrometry and protein identification

Bands belonging to FXa complexes in the sample incubated with DiES were excised manually from the gel and analysed by liquid chromatography and tandem mass spectrometry (LC-MS/MS). The proteomic analyses were performed at the proteomics facility of the Servei Central de Suport a la Investigació Experimental (SCSIE) of the University of Valencia (Spain). Samples were digested with sequencing grade trypsin (Promega) as Shevchenko *et al*. (1996) previously described. The digestion mixture was dried in a vacuum centrifuge and re-suspended in 20 *μ*L of 2% acetonitrile (ACN) and 0.1% trifluoroacetic acid (TFA). For LC-MS/MS, 5 *μ*L of each sample were loaded onto a column (NanoLC Column, 3 *μ* C18–CL, 350 *μ*m × 0.5 mm; Eksigent) and desalted with 0.1% TFA at 3 *μ*L min^−1^ for 5 min. Then, the peptides were loaded onto an analytical column (LC Column, 3 *μ* C18–CL, 75 *μ*m × 12 cm; Nikkyo) equilibrated in 5% ACN, 0.1% formic acid (FA). Elution was carried out with a linear gradient of 35% B in A (A: 0.1% FA; B: ACN, 0.1% FA) at a flow rate of 300 nL min^−1^ for 15 min. Peptides were analysed in a mass spectrometer nanoESIqQTOF (5600 Triple TOF, AB Sciex). Samples were ionized applying 2.8 kV to the spray emitter and analysed in a data-dependent mode. Survey MS1 scans were acquired from 350–1250 m z^−1^ for 250 ms. The quadrupole resolution was set to UNIT for MS2 experiments, which were acquired from 100–1500 m z^−1^ for 50 ms in a high sensitivity mode. Up to 25 ions were selected for fragmentation after each survey scan. Dynamic exclusion was set to 15 s. The system was controlled with 2 fmol of six proteins (LC Packings). Protein Pilot default parameters were used to generate peak list directly from 5600 TripleTof files. The Paragon algorithm (Shilov *et al*., 2007) of Protein Pilot v 4.5 was used to search the National Center for Biotechnology Information (NCBI) database.

The deduced amino-acid sequences of the resulting proteins of the LC-MS/MS analysis with an interesting function for the study were analysed using the following bioinformatic tools: BLAST searching of the homologous sequences in the NCBI and Swissprot/Uniprot databases (http://www.ncbi.nlm.nih.gov/, http://www.uniprot.org/), analysis of conserved protein domains with Prosite (https://prosite.expasy.org/), prediction of signal peptides with SignalP 3.0 (Bendtsen *et al*., 2004) (http://www.cbs.dtu.dk/services/SignalP) and multiple sequence alignment with ClustalW 2.1 (http://www.ebi.ac.uk/Tools/msa/clustalw2/).

### Statistical analysis

The results of the anticoagulation assays and the FXa inhibition assay were analysed with the Student's *t*-test. All the results were expressed as the mean ± the standard deviation (s.d.) of three independent experiments. In all of them, significant differences were defined as a *P* value <0.05 for a confidence level of 95%.

## Results

### DiES possess anticoagulant activity

**Fig. 1.**
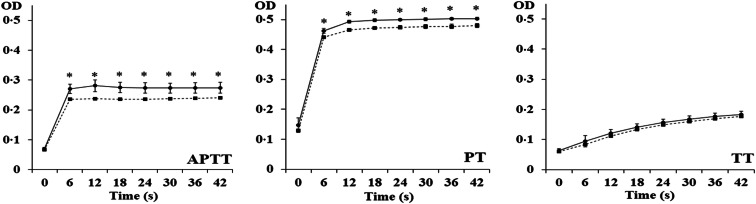
Anticoagulant activity of DiES evaluated by measuring the APTT, the PT and the TT. Plasma from healthy dogs was incubated with 0.5 *μ*g of DiES (■) or with PBS as negative control (●), and the corresponding reagent (APTT, PT or TT). Each point is the mean of three replicates ± s.d.. The asterisk (*) indicates significant differences (*P* < 0.05).

To study if DiES possessed anticoagulant activity, three clotting time assays were carried out. The APTT and the PT were prolonged by DiES since these assays showed that the clot was not completely formed in the presence of the antigenic extract, reaching optical densities significantly lower (*P* < 0.05) than those obtained by the control wells. Comparing both times, the obtained differences were slightly higher in the case of the APTT assay. No significant differences were found between groups in the TT assay (*P* < 0.05) (Fig. 1). These results determine that the modification of the coagulation cascade by DiES is produced against some factor/factors of the intrinsic, extrinsic or common pathways before transformation of fibrinogen into fibrin since the activity of fibrinogen is not altered by the antigenic extract.

### DiES inhibit FXa

**Fig. 2.**
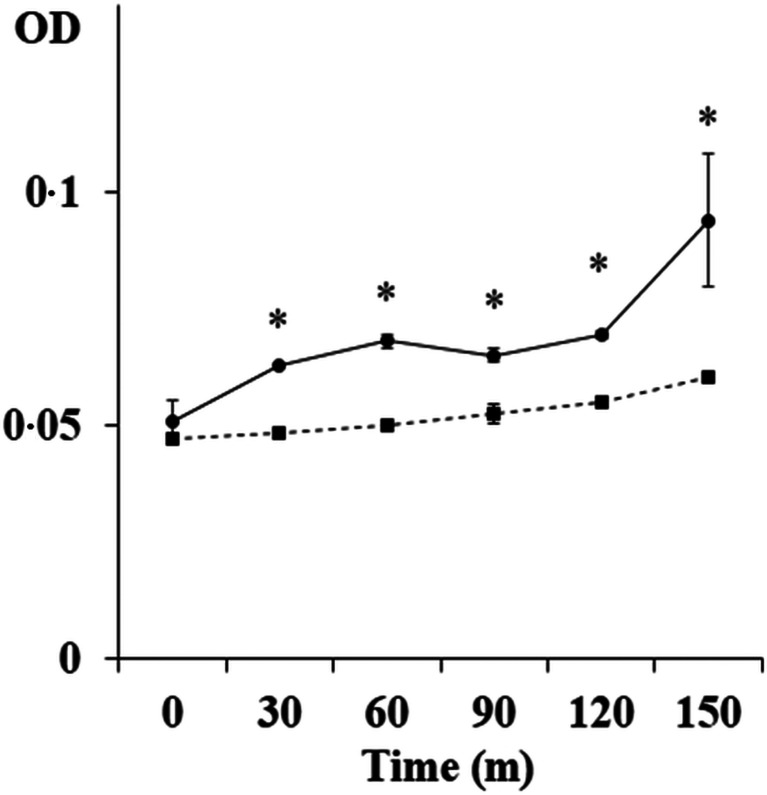
Inhibition of FXa by DiES. (■) 0.1 *μ*g of DiES were incubated with 4 nm FXa and 200 *μ*m S-2765 in a total volume of 100 *μ*L. (●) The presence of DiES was omitted and substituted by PBS as negative control in the previous reaction mixtures. Each point is the mean of three replicates ± s.d. The asterisk (*) indicates significant differences (*P* < 0.05).

In order to study the inhibitory effect of DiES against FXa, a chromogenic assay was carried out. The results showed that the factor did not completely hydrolyse the substrate in the presence of DiES since the optical densities were significantly lower than in the control wells over time (*P* < 0.05) (Fig. 2). This fact reveals that the activity of FXa is inhibited by some protein/proteins present in DiES.

### DiES proteins bind to FXa

**Fig. 3.**
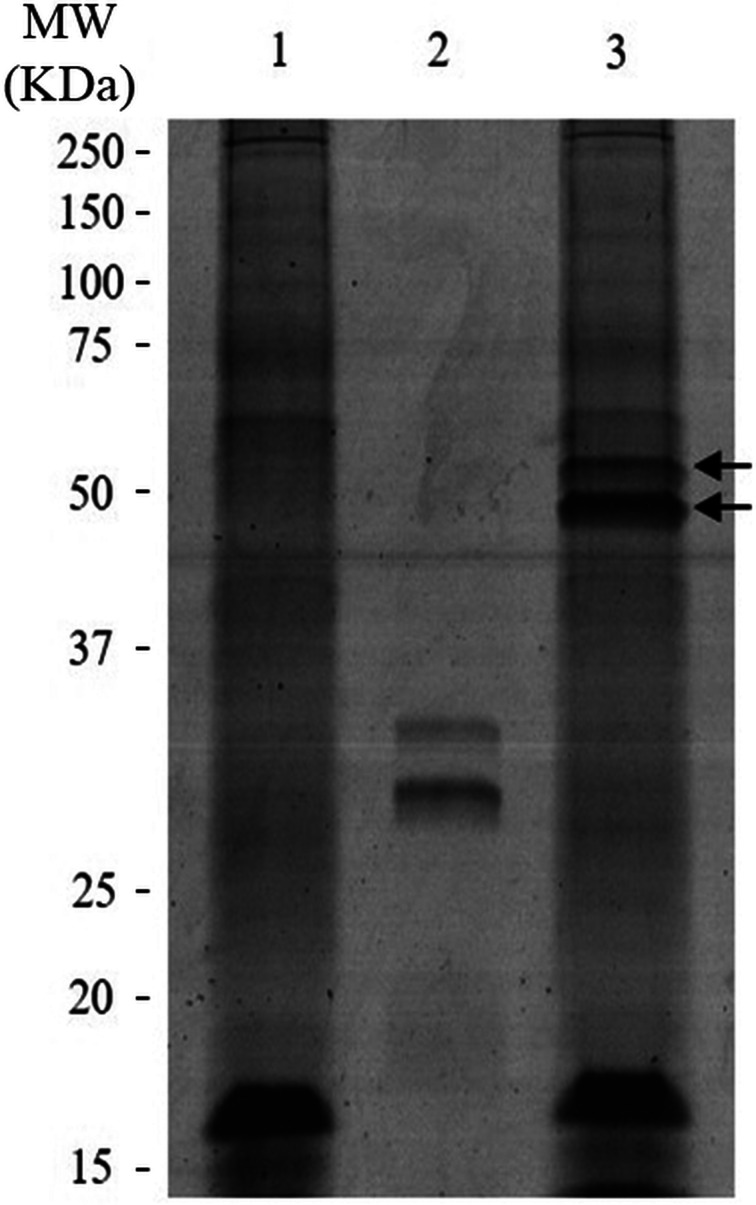
Binding of DiES to FXa. SDS-PAGE with 20 *μ*g of DiES (1), 1 *μ*g of FXa (2) and 20 *μ*g of DiES + 1 *μ*g of FXa (3) incubated in 5 mm Hepes buffer, pH 7.4. The two arrows mark the bands belonging to FXa in the sample incubated with DiES. The reference of the molecular weight pattern is indicated on the left.

A SDS-PAGE was carried out to determine if some protein of DiES was capable of binding to FXa into an irreversible complex. The electrophoresis gel (Fig. 3) revealed two bands in the lane containing the pre-incubated mixture of DiES and FXa that were absent in the lane containing only the antigenic extract. These bands, belonging to FXa, appeared in a larger molecular weight (50 and 56 kDa) than in the sample prepared only in the presence of FXa, in which they were respectively located at 30.5 and 33.5 kDa. These results suggest a complex formation between the coagulation factor and some protein of DiES.

### Identification of possible inhibitors of FXa in DiES

The two bands corresponding to FXa in the sample incubated with the antigenic extract (Fig. 3) were excised from the gel and identified by LC-MS/MS. In both of them, among different parasite proteins, the *Homo sapiens* coagulation factor X and the serpin protein 6, as a possible candidate to form an irreversible complex with FXa and inhibit its activity, were identified. Serpin protein 6 was identified by its similarity to the homologous protein from the filarial worm *Brugia malayi* (accession number: XP_001900434.1). These proteins were identified with a confidence percentage of 99% according to the equation [ProtScore = −log(1-(percent confidence/100))] in the Protein Pilot.

**Fig. 4.**
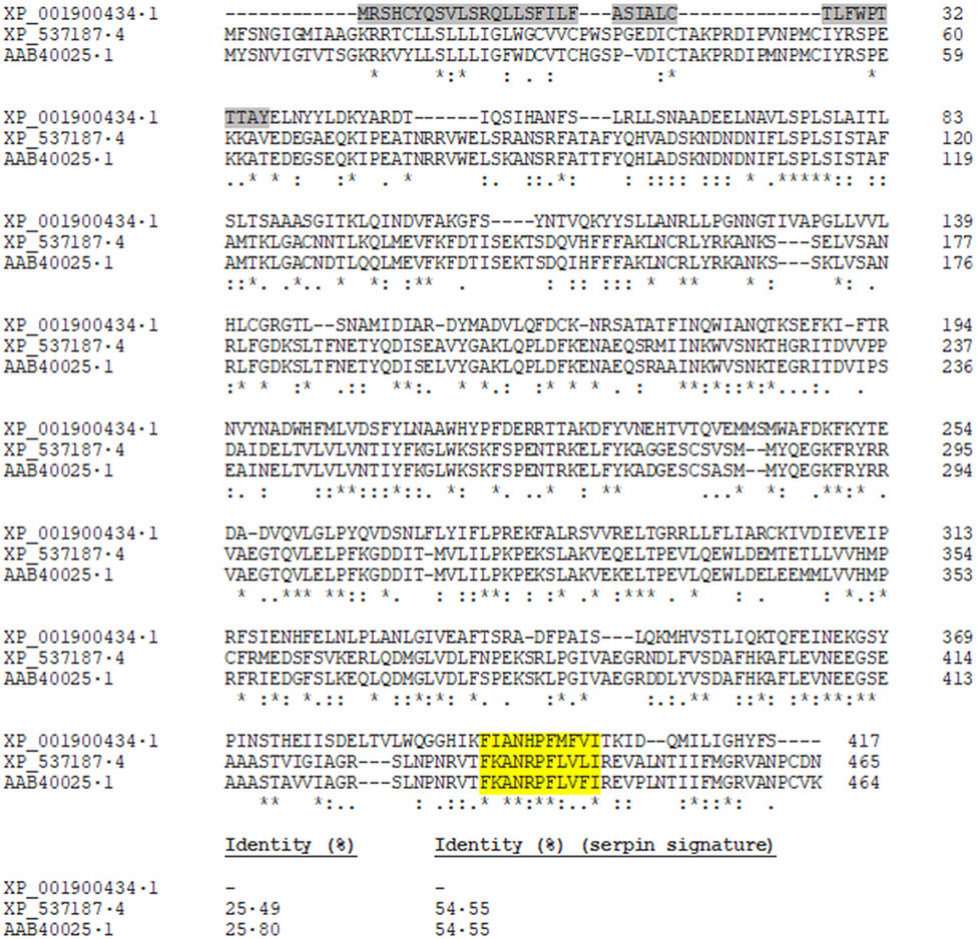
Alignment of the serpin protein 6 of *B. malayi* (XP_001900434.1) with the antithrombin III of *C. lupus familiaris* (XP_537187.4) and *H. sapiens* (AAB40025.1). The amino acids conserved in all the sequences are labelled with asterisks, and conservative and semiconservative substitutions are labelled with two and one point, respectively. The percentage of identity of the whole sequences, as well as the serpin signatures between *B. malayi* sequence and the others is indicated. The predicted signal peptide is shaded in grey, and the serpin signatures highlighted in yellow.

The bioinformatics analyses of the deduced amino acid sequence of serpin protein 6 revealed a signal peptide with the cleavage site predicted between positions 35 and 36, as well as a serpin signature domain (393–403) ‘FIANHPFMFVI’. Serpin protein 6 was also compared with the physiological inhibitor of FXa in mammals, the antithrombin III from *Canis lupus familiaris* and *H. sapiens* (accession numbers: XP_537187.4 and AAB40025.1) by a multiple sequence alignment. The analysis revealed an identity percentage of 25% between filarial and mammalian sequences, which increased up to 54.55% when the serpin signature domain was compared separately between sequences (Fig. 4).

## Discussion

Parasites have evolved to colonize their hosts and to survive in hostile environments such as the circulatory system. This would be related to the development of finely regulated mechanisms to confront and evade the responses of their hosts through the action of their metabolic products (excretory/secretory antigens) (Lightowlers and Rickard, 1988; Dzik, 2006; González-Miguel *et al*., 2016). These responses include the mechanisms of the blood coagulation system, which plays an important role as a barrier against foreign bodies, including blood parasites (Arneth, 2019). In this scenario, in which local thrombosis is part of the first line of host defence, the release of antigenic molecules capable of interacting with the haemostatic system can suppose a survival mechanism for this type of pathogen (Sun, 2006).

The objective of the present work was to analyse the ability of the excretory/secretory extract of the blood parasite *D. immitis* to modify the activity of the coagulation cascade of its host. This route is composed of a succession of enzymatic reactions involving the activation of zymogens in their catalytically active serine proteases and ending with the formation of the filamentous protein fibrin. Depending on the triggering phenomena, there are two major pathways in the blood clotting cascade, the intrinsic and the extrinsic pathways (Smith *et al*., 2015). The activity of these pathways is measured by the APTT and PT, respectively. Our results showed that the presence of DiES alters both times in blood samples from healthy dogs. On the other hand, the addition of DiES did not significantly modify the TT, which measures the time to convert fibrinogen to fibrin. Taken together these data suggest that *D. immitis* possesses in its excretory/secretory antigen molecules capable of inhibiting coagulation before the transformation of fibrinogen into fibrin. Similarly, anticoagulant activities have been identified in secretory products of a great variety of nematode and trematode helminth parasite species that spend at least part of their life cycle in close contact with the circulatory system of their hosts (Crawford *et al*., 1982; Foster *et al*., 1991; Cappello *et al*., 1995; Perteguer *et al*., 1996; Joachim *et al*., 2003; Yi *et al*., 2010; Mebius *et al*., 2013). This fact suggests that regulation of the coagulation cascade of the host through the secretion of molecules with inhibitory capacity is of paramount importance for blood parasites.

Regarding coagulation cascade, the intrinsic and the extrinsic pathways come to a common route at the level of inactivated factor X, which, once activated, forms FXa (Butenas and Mann, 2002). Our data demonstrated the ability of DiES to inhibit this coagulation factor suggesting that *D. immitis* secretes molecules with the capacity to modify the activity of FXa. Interestingly, FXa is one of the main target enzymes of the anticoagulant secreted molecules characterized in the parasitic groups (hookworms and ticks) whose interaction with the haemostatic host system has been studied more widely (Stassens *et al*., 1996; Harrison *et al*., 2002; Gan *et al*., 2009; Chmelař *et al*., 2017). The activity of this factor is key to the progression of haemostasis, playing a pivotal role in the blood coagulation cascade since it catalyses the production of thrombin as part of the prothrombinase complex with the cofactor, activated factor V, on the membrane surface (Butenas and Mann, 2002). Therefore, it has been proposed as a target for the development of new anticoagulant agents (Núñez-Navarro *et al*., 2019). This coagulation factor, as the proteolytic enzymes operating in the coagulation cascade, is all trypsin-like serine proteases, so that their activity can be inhibited by inhibitors of serine proteases or serpins (Nar, 2012). Serpins are a superfamily of key regulatory proteins involved in essential extracellular functions such as coagulation, fibrinolysis, complement activation and inflammation in mammals (Gettins, 2002; Lucas *et al*., 2018). The presence of serpins has been demonstrated in a large number of helminth parasites, in which they not only carry out endogenous physiological and regulatory functions, but their association with the vertebrate blood coagulation cascade, as well as with host immune modulation and/or evasion processes have been postulated (Zang and Maizels, 2001; Molehin *et al*., 2012). As serpins are irreversible suicide inhibitors, their mechanism of action against the various coagulation factors involves the formation of SDS-stable covalent inhibitory complexes with their target proteins (Gettins, 2002). In order to identify possible FXa-serpin inhibitory complexes in DiES we made a SDS-PAGE experiment, following by a mass spectrometry approach, allowing us to identify a *D. immitis* FXa inhibitor by its similarity to the homologous serpin 6 from *B. malayi*. Bioinformatic analysis of the resulting sequence defined the presence of a signal peptide and a conserved serpin signature domain. The signal peptide corroborates its secretion, which is important since many of this type of inhibitors are expressed on the surface of the parasite or excreted in its metabolic products, which would facilitate their anticoagulant action at the host–parasite interface (Knox, 2007). Regarding serpin signature domain, although nematode serpins have low overall homology to the serpins from mammalian species, their sequences are identical or conserved at most of the key amino acid positions (Zang and Maizels, 2001). This fact is consistent with the multiple sequence alignment carried out with the canine and human antithrombin III, which appears to be the most quantitatively important coagulation inhibitor in mammals, being to effectively neutralize all serine proteases produced during the blood coagulation process (Olson *et al*., 1993; Butenas and Mann, 2002). In this sense, our bioinformatic results demonstrated that serpin 6 sequence has a low homology to the mammalian serpins, which increases considerably when only the serpin signature domain is subjected to sequence alignment (Fig. 4).

In previous work carried out by our group we demonstrated the ability of *D. immitis* to activate the fibrinolytic system of the host by the presence in its excretory/secretory and cuticle extracts of numerous molecules with ability to bind plasminogen, enhance the generation of plasmin and stimulate the vascular endothelium to enhance the expression of physiological plasminogen activators [tissue plasminogen activator and the urokinase-type plasminogen activator, as well as decrease of the main fibrinolytic inhibitor (the plasminogen activator inhibitor-1)] (González-Miguel *et al*., 2012, 2013, 2015). The results presented in this work are coherent with the published data as they reinforce the ability of *D. immitis* to manipulate the haemostatic system of its host, not only through its pro-fibrinolytic activity, but also with its anticoagulant potential. A large number of strategies used by *D. immitis* to interfere with the haemostatic host system reflect the complexity of host–parasite relationships in cardiopulmonary dirofilariosis, as has been postulated in other blood parasites such as schistosomes or ticks (Mebius *et al*., 2013).

Regarding clinical aspects of cardiopulmonary dirofilariosis, a pro-coagulant state is one of the typical characteristics of the acute presentation of the pathology, but it triggers with the death of worms, either spontaneously or by treatment with filaricides, which immediately threaten the survival of the affected animal (Venco, 2007; Simón *et al*., 2012). In contrast, the chronic progression of the disease that is characterized by the presence of the live worms in the pulmonary arteries, apparently elapses without the appearance of serious thromboembolisms (Venco, 2007; González-Miguel *et al*., 2015). Therefore, the results obtained in the present work would be consistent with the apparent control in clot formation that accompanies cardiopulmonary dirofilariosis during its chronic progression, possibly influenced by the anticoagulant and pro-fibrinolytic molecules secreted by live worms in their metabolic products. On the one hand, this could suppose a survival mechanism for *D. immitis*, not only from the haemostatic point of view, but also as a strategy for evasion of the host immune response, which would be of great importance in a disease such as cardiopulmonary dirofilariosis, with an important inflammatory component at the vascular level. Recent studies have shown links between inflammation and coagulation, as well as an overlap between immunity and haemostasis providing evidence on the possible evolutionary path preceding the coagulation and immune systems (Witkowski *et al*., 2016; Antoniak, 2018; Arneth, 2019). On the other hand, the alteration of the haemostatic balance of the host due to the effect of parasitic antigens could have a pathological effect (González-Miguel *et al*., 2015). Paradoxically, hyperfibrinolysis can result in bleeding, and may be based on dysregulation at either the cell surface or in the fluid phase. One of the most common hyperfibrinolytic states is disseminated intravascular coagulation (DIC), where systemic inflammation results in increased levels of fibrin in microthrombi. This consumptive coagulopathy leads to a deficiency of circulating fibrinogen and increased bleeding tendencies (Chapin and Hajjar, 2015). DIC may be present in *Dirofilaria* infected dogs with heavy parasite loads in their final locations (Strickland, 1998), and it is accompanied by an increase in plasma levels of d-dimer, (a fibrin degradation product) that have been detected in dogs with dirofilariosis compared with healthy controls (Carretón *et al*., 2011).

The data presented in this work reveal, for the first time, the anticoagulant capacity of the excretory/secretory antigens from the blood parasite *D. immitis*, which could be related to the control of the formation of clots in its intravascular habitat and, therefore, it could represent a mechanism that facilitates its survival in the circulatory system of its host. Future research will allow us to identify and characterize these molecules, which could be important not only as targets in the control of cardiopulmonary dirofilariosis, but also as potential new drugs in the treatment of haemostatic disorders.
